# Supplementary material for: IL-17+ CD8+ T cell suppression by dimethyl fumarate associates with clinical response in multiple sclerosis
Source: Nat Commun. 2019 Dec 16;10:5722. doi: 10.1038/s41467-019-13731-z (PMC6915776; doi:10.1038/s41467-019-13731-z)
Supplement: Supplementary file 1 — Supplementary Information [file 41467_2019_13731_MOESM1_ESM.pdf]

IL-17$^+$ CD8$^+$ T cell suppression by dimethyl fumarate associates with clinical response in multiple sclerosis

Christina Lückel[1,2*], Felix Picard[1*], Hartmann Raifer[1,3*], Lucia Campos Carrascosa[1,4], Anna Guralnik[1], Yajuan Zhang[1], Matthias Klein[2], Stefan Bittner[5], Falk Steffen[5], Sonja Moos[6], Federico Marini[7,8], Renee Gloury[9,10], Florian C. Kurschus[6,11], Ying-Yin Chao[12], Wilhelm Bertrams[13], Veronika Sexl[14], Bernd Schmeck[13,15], Lynn Bonetti[16], Melanie Grusdat[16], Michael Lohoff[1], Christina E. Zielinski[12], Frauke Zipp[5], Axel Kallies[9,10], Dirk Brenner[16,17], Michael Berger[18], Tobias Bopp[2,19], Björn Tackenberg[20] and Magdalena Huber[1]

Supplementary Information

1. **Supplementary Figures 1-9.**
2. **Supplementary Tables 1-7.**

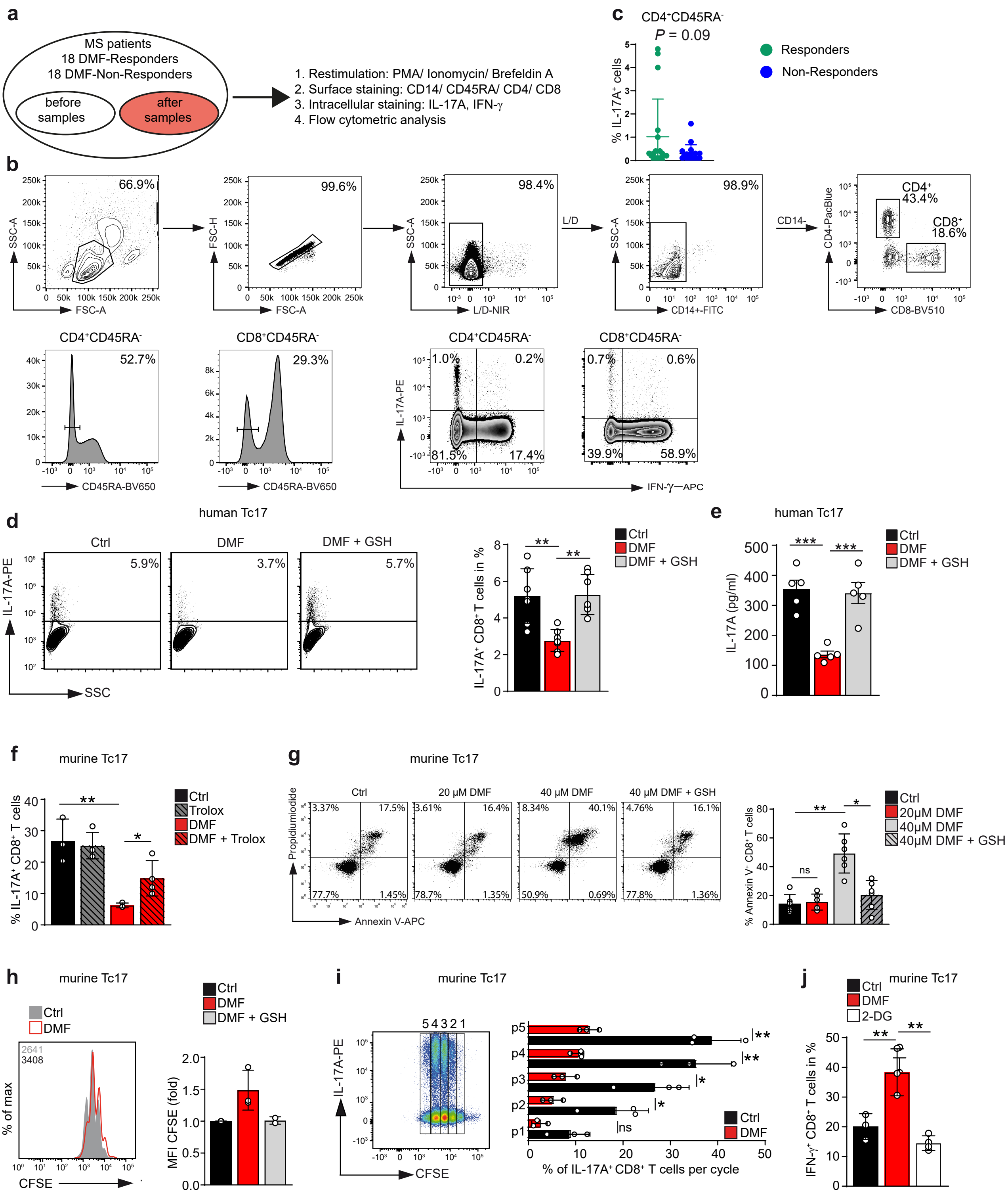

a
MS patients
18 DMF-Responders
18 DMF-Non-Responders
before samples
after samples
1. Restimulation: PMA/ Ionomycin/ Brefeldin A
2. Surface staining: CD14/ CD45RA/ CD4/ CD8
3. Intracellular staining: IL-17A, IFN-γ
4. Flow cytometric analysis
c
CD4+CD45RA-
P = 0.09
% IL-17A+ cells
Responders
Non-Responders
b
66.9%
99.6%
98.4%
98.9%
SSC-A
FSC-A
FSC-H
L/D-NIR
L/D
CD14-
CD14+-FITC
CD4-PacBlue
CD8-BV510
CD4+ 43.4%
CD8+ 18.6%
CD4+CD45RA-
52.7%
CD45RA-BV650
CD8+CD45RA-
29.3%
1.0%
0.2%
81.5%
17.4%
0.7%
0.6%
39.9%
58.9%
IL-17A-PE
IFN-γ—APC
d
human Tc17
Ctrl
DMF
DMF + GSH
5.9%
3.7%
5.7%
IL-17A-PE
SSC
IL-17A+ CD8+ T cells in %
e
human Tc17
IL-17A (pg/ml)
f
murine Tc17
Ctrl
Trolox
DMF
DMF + Trolox
% IL-17A+ CD8+ T cells
g
murine Tc17
Ctrl
20 μM DMF
40 μM DMF
40 μM DMF + GSH
3.37%
17.5%
77.7%
1.45%
3.61%
16.4%
78.7%
1.35%
8.34%
40.1%
50.9%
0.69%
4.76%
16.1%
77.8%
1.36%
Propidiumiodide
Annexin V-APC
% Annexin V+ CD8+ T cells
20μM DMF
40μM DMF
40μM DMF + GSH
ns
h
murine Tc17
Ctrl
DMF
2641
3408
% of max
CFSE
DMF + GSH
MFI CFSE (fold)
i
murine Tc17
5 4 3 2 1
IL-17A-PE
CFSE
p5
p4
p3
p2
p1
% of IL-17A+ CD8+ T cells per cycle
ns
j
murine Tc17
Ctrl
DMF
2-DG
IFN-γ+ CD8+ T cells in %

Supplementary Fig. 1. Characterization of DMF effects on Tc17 cells. **a**, Experimental setup for the processing of frozen PBMC samples “before” and “after” from the same donors fulfilling (Responders, n=18) or not (Non-Responders, n=18) NEDA-3 criteria after DMF therapy (Supplementary Table 1-3). Samples before and after (about one year) treatment initiation were thawed and restimulated. Subsequently, the samples were stained at the surface, then intracellularly and proceeded for the acquisition by flow cytometry and analysis (steps 1-4). **b**, General flow cytometry gating strategy for analysis of the frequencies of IL-17^{+} cells among CD45RA^{-}CD8^{+} as well as CD45RA^{-}CD4^{+} T cells. Acquired cells were first gated for exclusion of debris (FSC-H vs SSC-H), then for singlets (FSC-A vs FSC-H), viable cells were identified using Zombie-NIR and gating Zombie-NIR^{-} cells, then monocytes were excluded by gating on CD14^{-} cells. Memory CD4^{+} and CD8^{+} T cell subsets were identified by gating of CD45RA^{-}CD4^{+} or CD45RA^{-}CD8^{+} cells, in which IL-17 and IFN-γ positivity was analyzed. **c**, Frequency of CD4^{+}CD45RA^{-}IL-17A^{+} cells before DMF therapy in Responders (R, n=18) versus Non-Responders (N-R, n=18), p value evaluated by two-tailed, unpaired t-test **d**, **e**, CD45RA^{-}CD8^{+} T cells were sorted from healthy human peripheral blood (PB) and differentiated under type 17 conditions for 96h with indicated treatment (DMSO (Ctrl), DMF 10μM, DMF+GSH 50μM). **d**, Flow cytometry of IL-17A^{+}CD8^{+} T cells. **e**, ELISA determination of IL-17A in the supernatant from cultures described in (d). **f**, Flow cytometry of IL-17A in murine Tc17 cells differentiated for 72h, with indicated treatment (Trolox 400μM). **g**, Flow cytometry of apoptosis in murine Tc17 cells differentiated for 72h with indicated treatment after staining with Annexin V and propidium iodide. **h**, Flow cytometry of proliferation of murine Tc17 cells labeled with carboxyfluorescein succinimidyl ester (CFSE) and differentiated for 72h with indicated treatment. Bars show fold MFI change of CFSE normalized to control, which was arbitrarily set to 1. **i**, Flow cytometry of proliferation and IL-17A in Tc17 cells differentiated for 72h with indicated treatment after restimulation. Bars show percentages of IL-17A^{+}CD8^{+} T cells in each proliferation cycle. **j**, Flow cytometry of IFN-γ in Tc17 cells differentiated for 72h with indicated treatment (250μM 2-DG). Graphs show mean ±s.d. from three individual experiments and donors (d, n=7; e, n=5), or six (g) or three to four (f and h-j) combined experiments; (c, d-j) individual values are plotted. In (d-h and j), *p<0.05, **p<0.01, ***p<0.001, ****p<0.001 evaluated by one-way ANOVA followed by Tukey’s HSD multiple comparison test, in (i), *p<0.05, **p<0.01 by two-tailed, unpaired t-test.

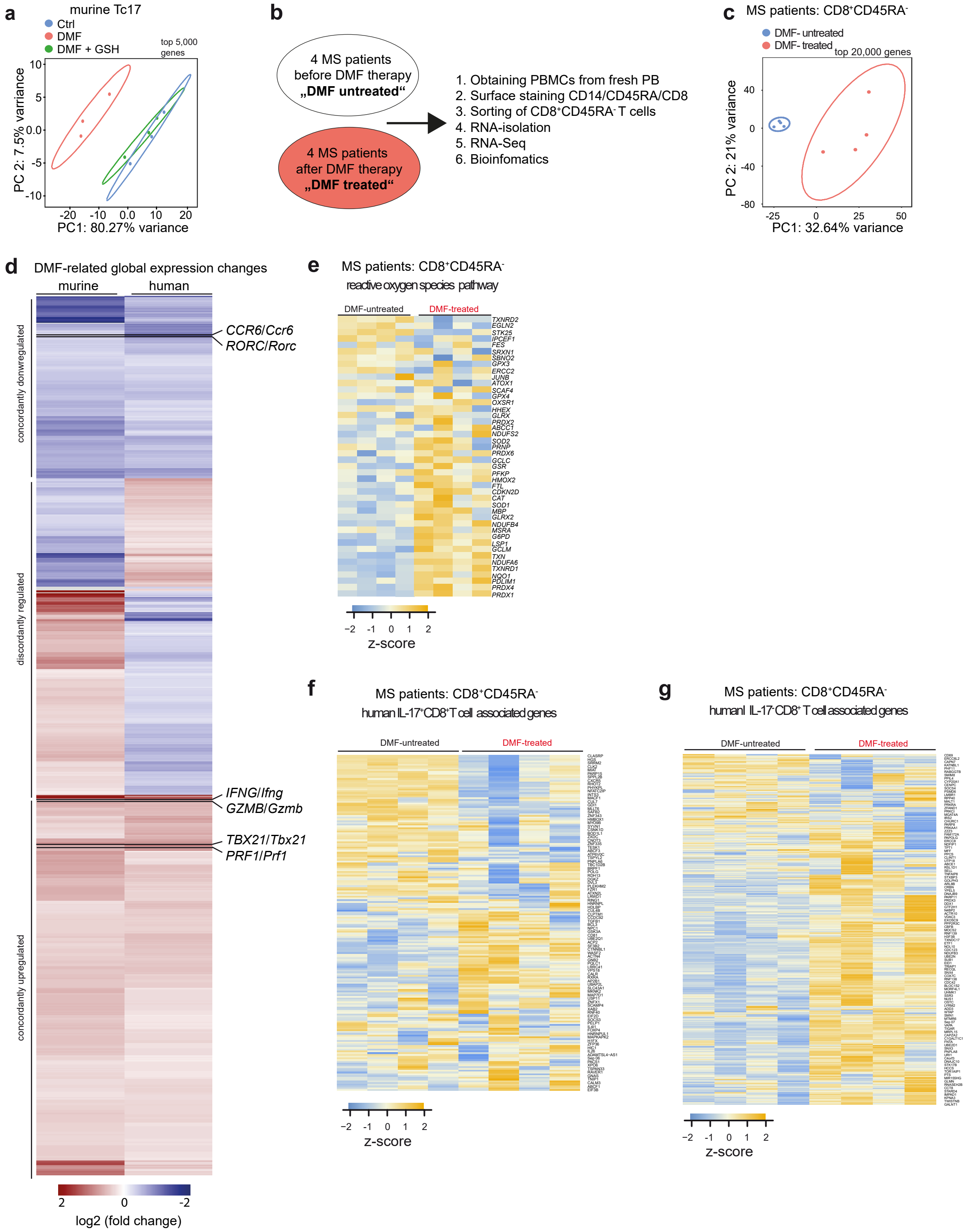
a
murine Tc17
Ctrl
DMF
DMF + GSH
top 5,000 genes
PC 2: 7.5% variance
PC1: 80.27% variance
b
4 MS patients before DMF therapy „DMF untreated“
4 MS patients after DMF therapy „DMF treated“
1. Obtaining PBMCs from fresh PB
2. Surface staining CD14/CD45RA/CD8
3. Sorting of CD8+CD45RA- T cells
4. RNA-isolation
5. RNA-Seq
6. Bioinfomatics
c
MS patients: CD8+CD45RA-
DMF- untreated
DMF- treated
top 20,000 genes
PC 2: 21% variance
PC1: 32.64% variance
d
DMF-related global expression changes
murine
human
concordantly donwregulated
discordantly regulated
concordantly upregulated
CCR6/Ccr6
RORC/Rorc
IFNG/Ifng
GZMB/Gzmb
TBX21/Tbx21
PRF1/Prf1
log2 (fold change)
e
MS patients: CD8+CD45RA-
reactive oxygen species pathway
DMF-untreated
DMF-treated
TXNRD2
EGLN2
STK25
IPCEF1
FES
SRXN1
SBNO2
GPX3
ERCC2
JUNB
ATOX1
SCAF4
GPX4
OXSR1
HHEX
GLRX
PRDX2
ABCC1
NDUFS2
SOD2
PRNP
PRDX6
GCLC
GSR
PFKP
HMOX2
FTL
CDKN2D
CAT
SOD1
MBP
GLRX2
NDUFB4
MSRA
G6PD
LSP1
GCLM
TXN
NDUFA6
TXNRD1
NQO1
PDLIM1
PRDX4
PRDX1
z-score
f
MS patients: CD8+CD45RA-
human IL-17+CD8+T cell associated genes
DMF-untreated
DMF-treated
z-score
g
MS patients: CD8+CD45RA-
human IL-17-CD8+ T cell associated genes
DMF-untreated
DMF-treated
z-score

Supplementary Fig. 2. DMF causes a comparable shift in genetic signatures in murine and human Tc17 cells. **a** Principal component analysis (PCA) of the top 5,000 differentially expressed genes from murine Tc17 cells with the indicated treatment (dataset from Fig. 2a). **b**, Experimental setup for the processing of fresh PB samples from patients before DMF therapy ("DMF-untreated" n=4) and matched patients fulfilling NEDA-3 criteria after 12-13 months of DMF therapy ("DMF-treated" n=4). From fresh PB, PBMCs were obtained, which were stained at the surface and subsequently sorted for memory $CD8^+$ T cells. RNA was isolated, sequenced and bioinformatically analysed (steps 1-6). **c**, PCA of top 20,000 DE genes in $CD8^+CD45RA^-$ T cells from matched MS patients before ("DMF-untreated" n=4) and after 12 months of DMF treatment fulfilling NEDA-3 criteria ("DMF-treated" n=4) based on RNAseq (dataset from Fig. 2f). **d**, Heatmap of rlog transformed expression values of changes in response to DMF in murine Tc17 cells (dataset from Fig. 2a) and human $CD8^+CD45RA^-$ T cells obtained from MS patients (dataset from Fig. 2f) (p adj<0.1). Highlighted are genes associated with Tc17 and CTL fate. **e**, Heatmap of color coded z-scores for the rlog transformed expression values based on the GSEA comparing the relative expression of genes in $CD8^+CD45RA^-$ T cells from "DMF-untreated" and matched "DMF-treated" MS patients examining the distribution of genes involved in ROS-signaling (MSigDBv6.1, hallmark dataset). **f**, **g**, Heatmap of color coded z-scores for the rlog transformed expression values based on the GSEA comparing the relative expression of genes in $CD8^+CD45RA^-$ T cells from "DMF-untreated" versus matched "DMF-treated" MS patients examining the distribution of $IL17^+CD8^+$ (f) or $IL17^-CD8^+$ (g) signature genes according to Mielke et al[42]

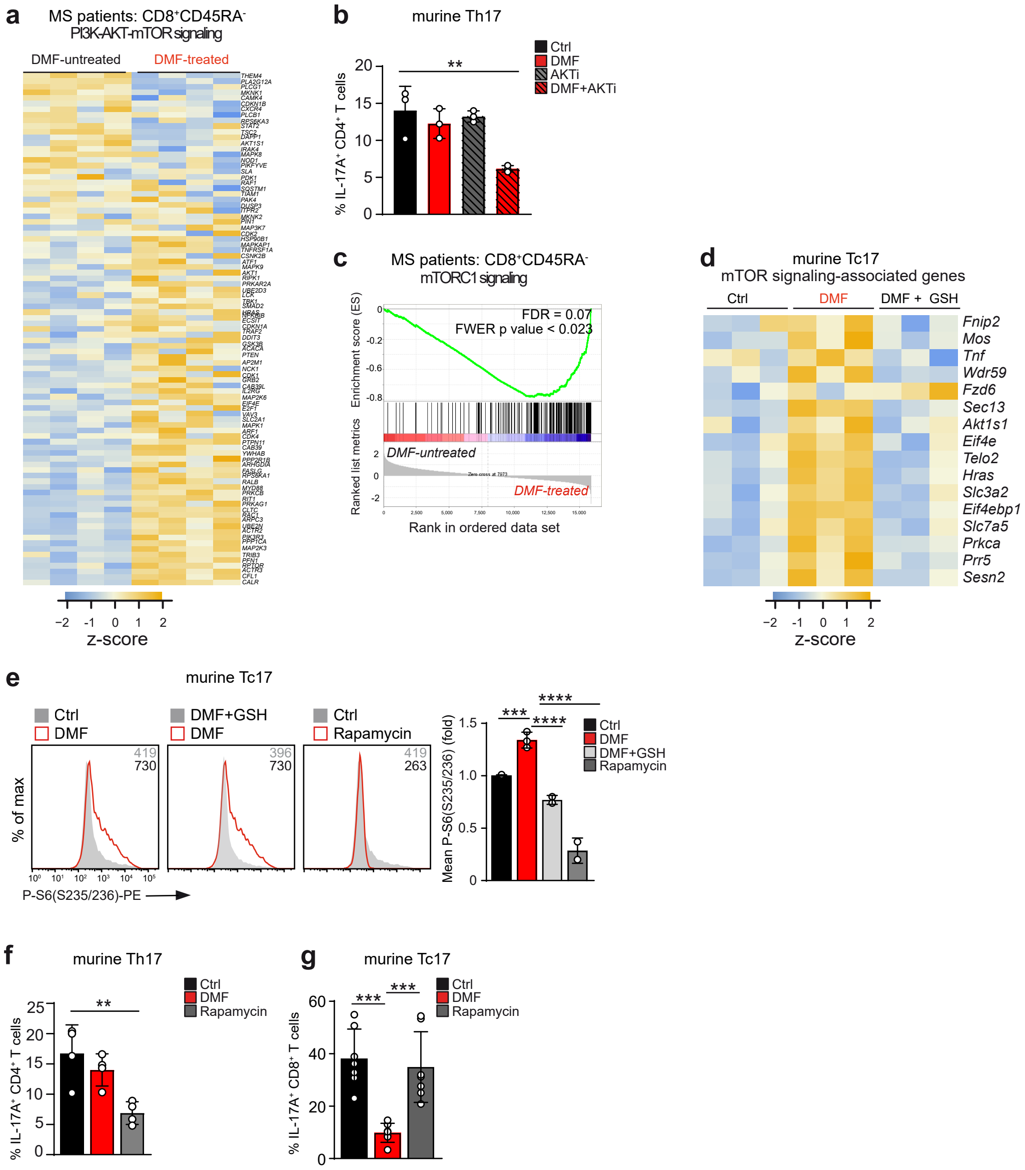

Supplementary Fig. 3. Differential impact of AKT- and mTOR-signaling on IL-17 production by Tc17 and Th17 cells. **a**, Heatmap of color-coded z-scores for the rlog transformed expression values based on the GSEA comparing the relative expression of genes in CD8$^+$CD45RA$^-$ T cells from matched group of MS patients, "DMF-untreated" and "DMF-treated", examining the distribution of genes involved in PI3K-AKT-mTOR-signaling (MSigDBv6.1, hallmark dataset), dataset from Fig. 2f. **b**, Flow cytometry of IL-17A in pathogenic Th17 cells differentiated for 72h with indicated treatment (AKTi 1µM). **c**, GSEA comparing the relative expression of genes in CD8$^+$CD45RA$^-$ T cells from matched group of MS patients, "DMF-untreated" and "DMF-treated", examining the distribution of genes involved in mTORC1 signaling (MSigDBv6.1, hallmark dataset, dataset from Fig. 2f). **d**, Heatmap showing the 16 most differentially upregulated mTOR-signaling-associated genes (KEGG database) in murine Tc17 (dataset from Fig. 2a). **e,** Flow cytometry of P-S6(S235/236) in murine Tc17 cells differentiated for 48h ±DMF, DMF+GSH, ±50 nM Rapamycin. Bars to the right show fold MFI normalized to the control, which was arbitrarily set to 1. **f**, **g**, Flow cytometry of IL-17A in pathogenic Th17 (**f**) or Tc17 (**g**) cells differentiated for 72 h with indicated treatment. Bars show mean ±s.d. from seven (**g**), four (**f**) or three (**b, e**) or combined experiments; individual values are plotted. In (**b, e-g**) **p<0.01, ***p<0.001, ****p<0.0001 evaluated by one-way ANOVA followed by Tukey's HSD multiple comparison test.

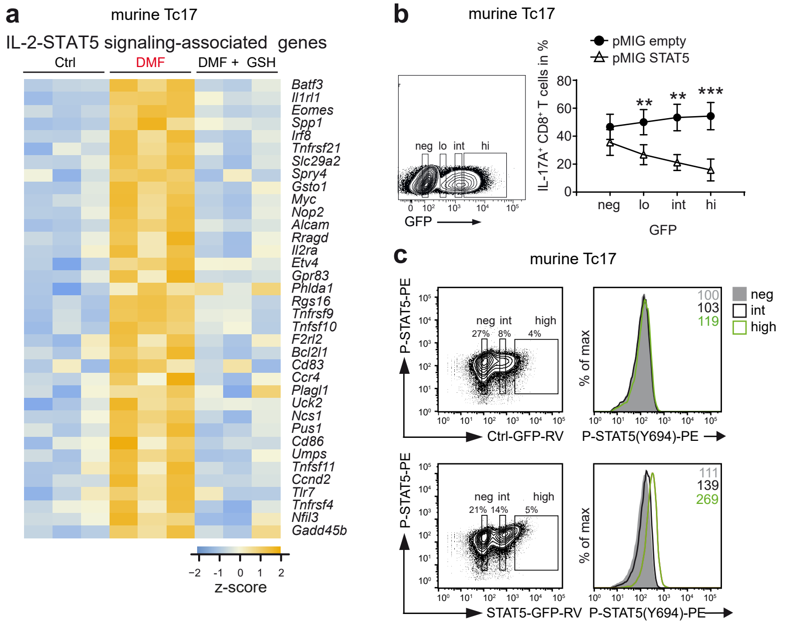

Supplementary Fig. 4. DMF suppresses IL-17 production in Tc17 cells via enhanced IL-2-STAT5 signaling. **a**, Heatmap of the rlog transformed, batch-corrected top 36 differentially expressed genes in IL-2-STAT5 signaling (GSEA hallmark dataset MSigDBv6.1) in murine Tc17 ±DMF or DMF+GSH dataset from (Fig. 2a). **b**, Flow cytometry of IL-17A$^+$GFP$^+$CD8$^+$ T cells. Murine Tc17 cells were retrovirally transfected with either constitutive active P-STAT5 (pMIG STAT5) or GFP alone vectors (pMIG empty). Contour-plot shows four subsets, based on the GFP expression intensity (GFP-negative, neg, GFP-low, lo, GFP-intermediate, int, and GFP-high, hi), which were analyzed for IL-17A (graph shown to the right, n=3). **c**, Flow cytometry of P-STAT5(Y694) in murine Tc17 cells after retroviral transduction with either pMIG STAT5 or pMIG empty. Dot plots show three subsets (GFP-negative, neg, GFP-intermediate, int, and GFP-high, hi). Data shows mean ±s.d. from four (b) combined experiments or one representative from three performed experiments (c). In (b) **p<0.01, ***p<0.001 evaluated two-tailed, unpaired t-test.

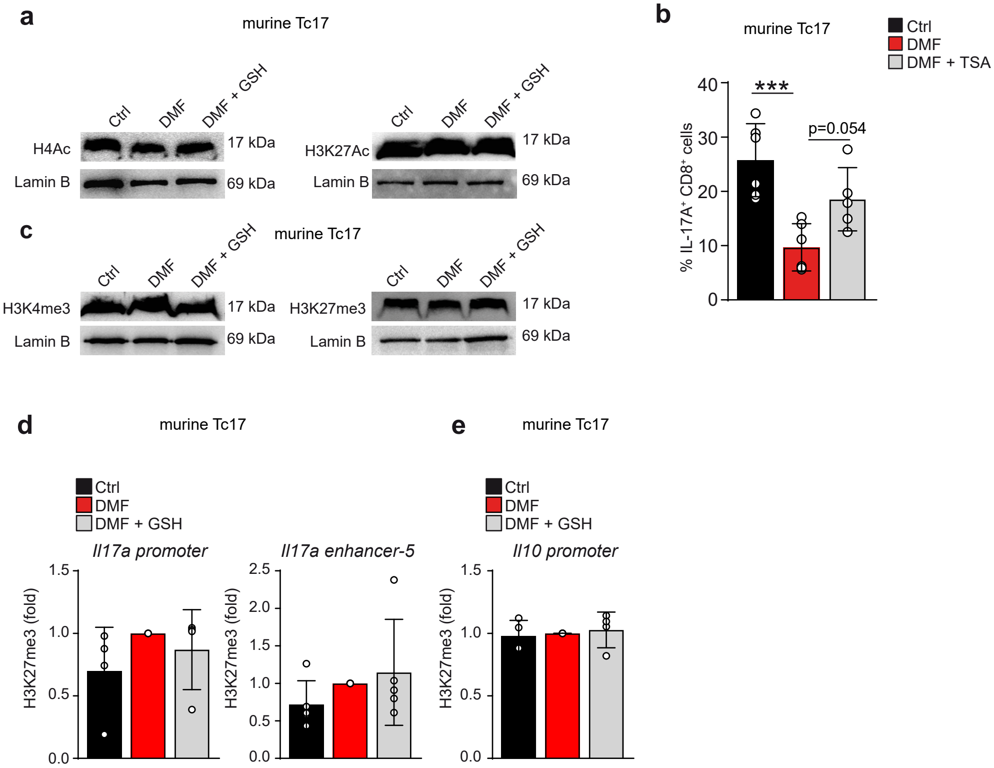

Supplementary Fig. 5. DMF does not alter global histone modifications in Tc17 cells. **a**, **c**, Immunoblot analysis of indicated histone modifications in murine Tc17 cells differentiated for 72h ±DMF or DMF+GSH. Data are representative of three independent experiments. **b**, Flow cytometry of IL-17A in murine Tc17 cells differentiated for 72h with indicated treatment (1nM TSA). **d**, ChIP assay for H3K27me3 at the *Il17* promoter and *Il17* enhancer-5 in murine Tc17 cells differentiated for 72h ±DMF or DMF+GSH. **e**, ChIP assay for H3K27me3 at the *Il10* promoter in murine Tc17 cells differentiated for 72h ±DMF or DMF+GSH. Bars show mean ±s.d. of fold change normalized to DMF treatment, which was arbitrarily set to 1. Data from four to six (b, d and e) combined experiments; individual values are plotted. In (b, d and e) significance evaluated by one-way ANOVA followed by Tukey's HSD multiple comparison test.

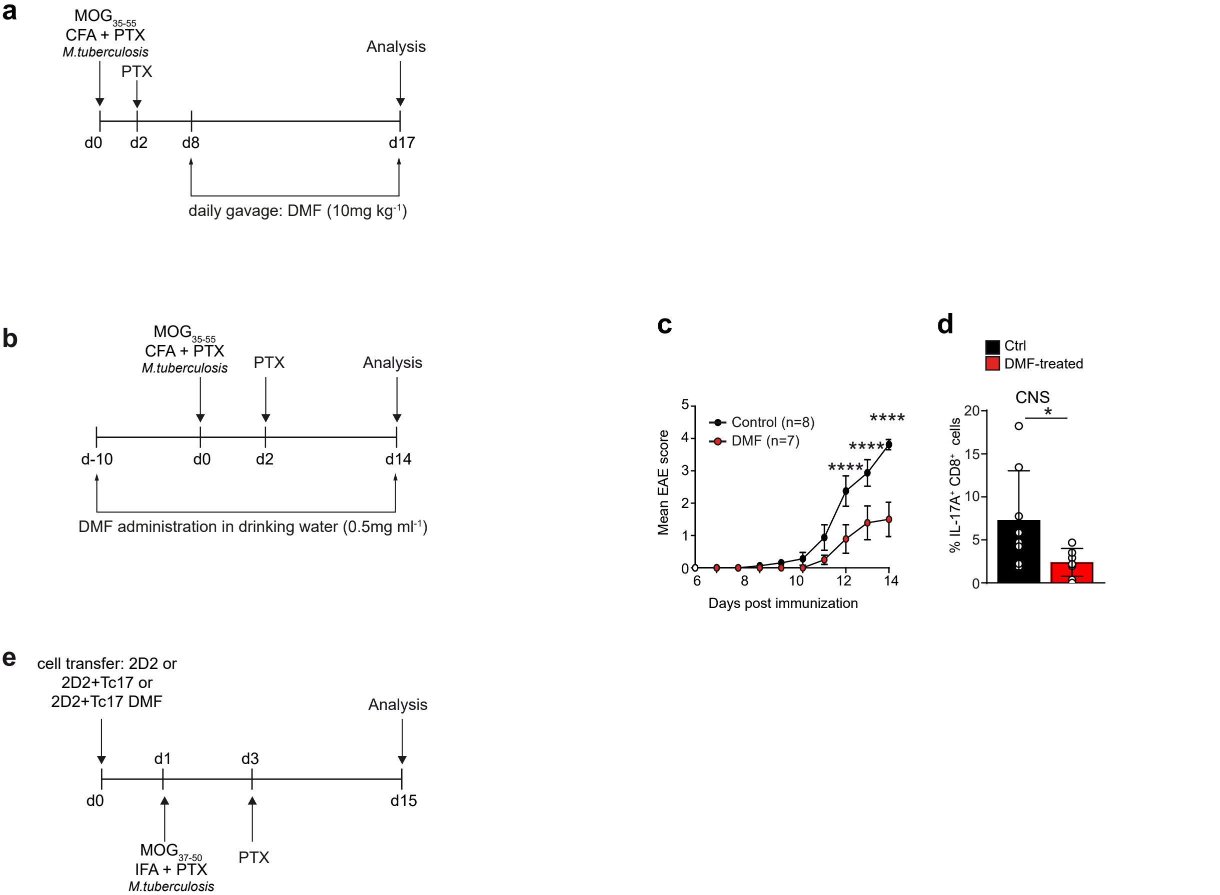

Supplementary Fig. 6. DMF limits pathogenicity of Tc17 cells in EAE. **a,b** Outline of experimental strategy. WT mice were immunized with MOG_{35-55}, CFA and M. tuberculosis for induction of EAE. Pertussis toxin (PTX) was injected i.p. along with immunization and on day 2 post immunization (p.i.). **a**, Therapeutic application of DMF since onset of clinical symptoms (d8) by daily oral gavage, either with 10mg kg^{-1} DMF in 0.6% Methocel or by vehicle (0.6% Methocel) alone. Analysis was performed on day 17 p.i. **b**, Preventive application of DMF in drinking water starting 10 days before EAE induction and during the course of disease. Analysis was performed on day 14 p.i. **c**, Mean clinical scores (±s.d.) of MOG_{35-55} immunized wild-type (WT) mice (n=8) with preventive DMF treatment. **d**, Percentages of IL-17A$^+$CD8$^+$ T cells (mean ±s.d.; n=8) in the CNS of WT mice ±DMF treatment. Individual values are plotted. **e**, Outline of experimental strategy for adoptive transfer EAE. *Irf4*$^{-/-}$ mice received 10^3 CD4$^+$ 2D2 cells alone or together with 2.5×10^6 Tc17 cells differentiated for 96 h ±DMF. EAE was induced on the next day by injection of MOG_{37-50}, IFA and M. tuberculosis. PTX was injected i.p. on day 0 and 2 p.i. Analysis was performed 15 days after cell transfer. In (c), ****p<0.0001 evaluated by two-way ANOVA with Bonferroni post-hoc test, in (d) *p<0.05 by two-tailed, unpaired t-test with Welch`s correction.

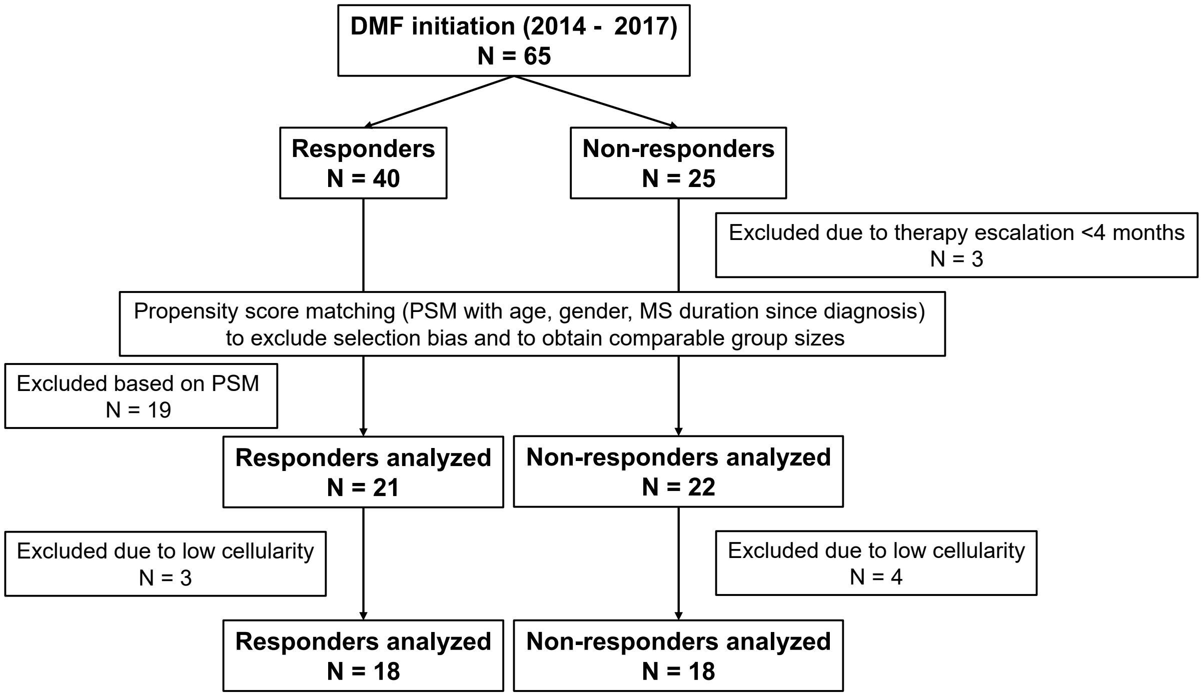

Supplementary Fig. 7. Scheme of study design for collection of DMF therapy responders and non-responders. In total, 65 MS patients were included in the study cohort, compromising 40 responders and 25 non-responders. 3 non-responders were excluded because of early therapy escalation. Propensity score matching was performed with the variables age, gender, MS duration since diagnosis, and EDSS before treatment to select 21 out of 40 patients yielding in comparable group sizes for responders and non-responders. Selected (n = 21) and excluded (n = 19) responders did not differ concerning above mentioned parameters. From the measured 21 DMF-responder samples, 3 samples were excluded while from 22 non-responder samples, 4 samples were excluded, all because of low cellularity.

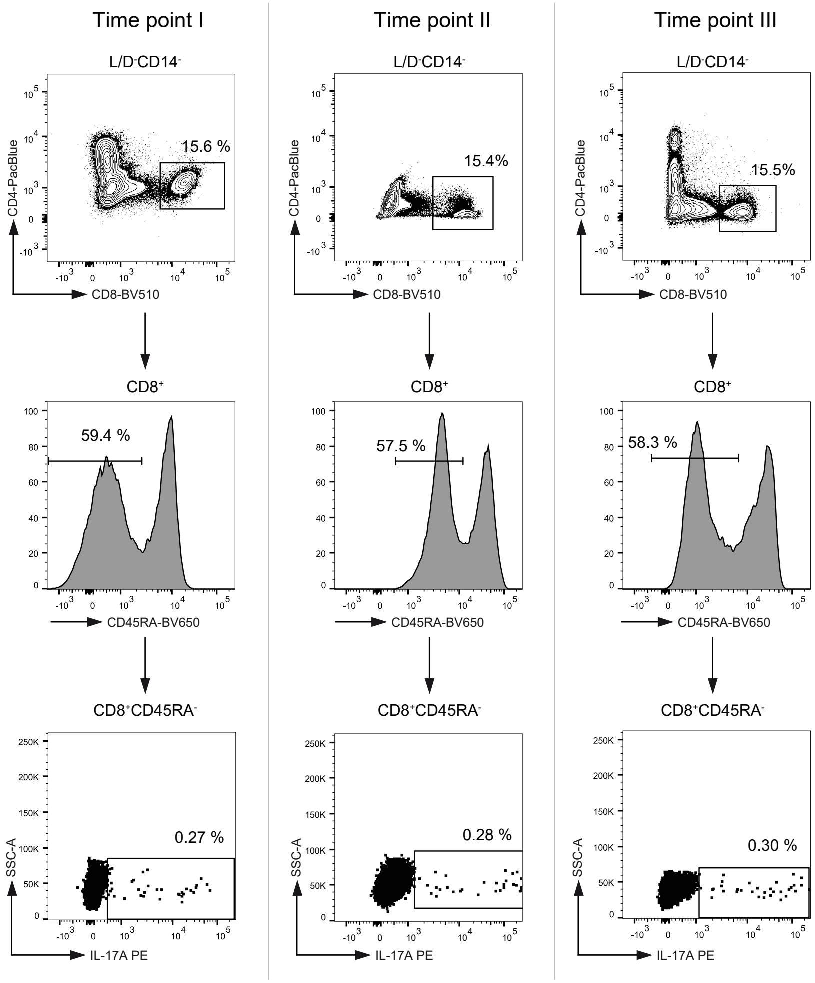

Supplementary Fig. 8. Flow cytometry batch effect validation. Blood from a healthy donor was drawn, processed for PBMC isolation and then frozen in several aliquots which served as control samples. The control samples were thawed, rested, restimulated, fixed, stained, acquired and analyzed on three different time points: at the start of responder sample analysis (time point I) and one year later at two different days (time points II and III, for non-responder analysis) using the same standard protocols by the same person. In the top panel, gating for $CD8^+$ T cells is depicted, in the middle, gating of memory $CD45RA^-CD8^+$ T cells and at the bottom, IL-17^+ cells. Arrows indicate the sequential gating strategy. Numbers indicate percentages of gated cells.

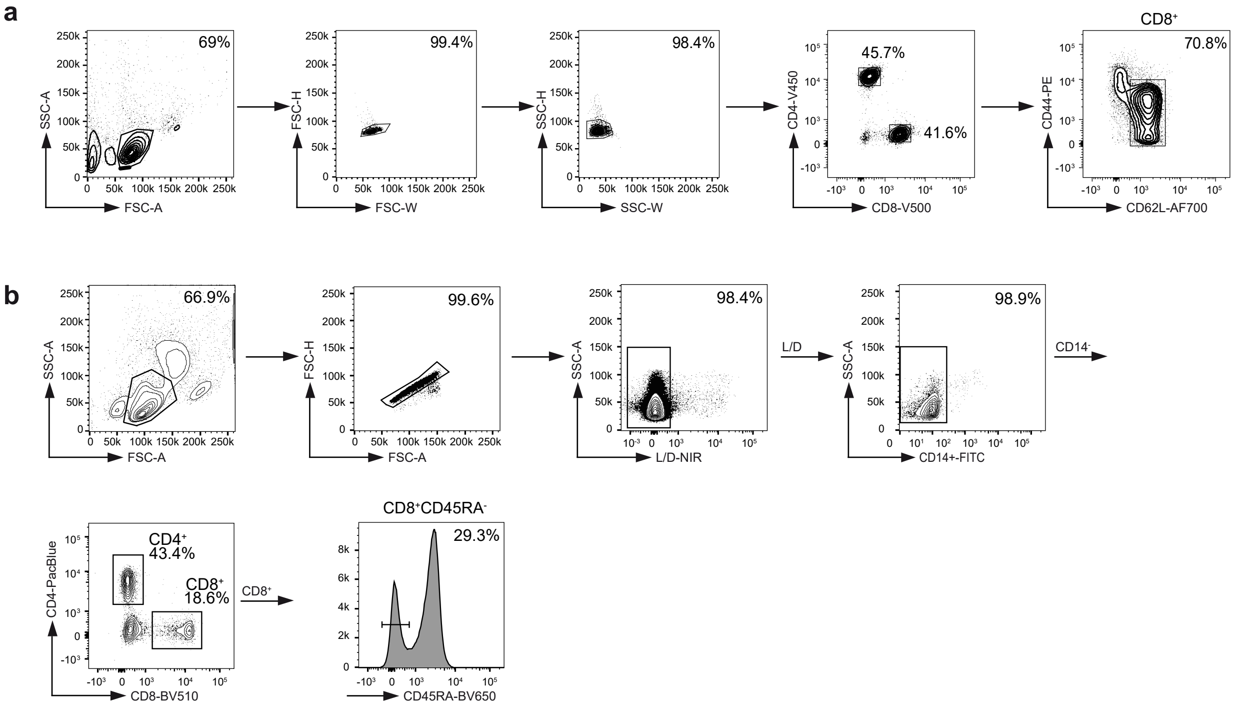

Supplementary Fig. 9. Exemplary gating strategies for sorting. **a**, General flow cytometry gating strategy for sorting murine naive CD4$^+$ or CD8$^+$ cell populations. T cells were enriched using Miltenyi negative selection kits. Acquired cells were first gated for exclusion of debris (FSC-A vs SSC-A), then for singlets (SSC-H vs SSC-W or FSC-H vs FSC-W), then either CD4$^+$ or CD8$^+$ cells were identified by gating on CD4 vs CD8 and the respective naive CD62$^+$CD44$^-$ subpopulation was sorted from the CD44 vs CD62L subgate. **b**, General flow cytometry gating strategy for sorting human CD45RA$^-$CD8$^+$ as well as CD45RA$^-$CD4$^+$ T cells for further RNAseq analysis. Acquired cells were first gated for exclusion of debris (FSC-A vs SSC-A), then for singlets (FSC-A vs FSC-H), viable cells were identified using Zombie-NIR and gating Zombie-NIR$^-$ cells, then monocytes were excluded by gating on CD14$^-$ cells. CD8$^+$ cells were identified by gating on CD4 vs CD8. Memory CD8$^+$ T cell subsets for sorting were identified by gating on CD45RA$^-$CD8$^+$ cells.

Supplementary Tables 1-7.

Supplementary Table 1. Characteristics of MS patient cohort included for IL-17A analysis (Fig. 1a, b, Supplementary Fig. 1a-c). Upon DMF therapy we distinguished between Responders (NEDA-3 positive) and DMF Non-Responders (NEDA-3 negative) MS patients.

**Clinical data**	**Responders (n=18)**	**Non-Responders (n=18)**
Age at MS[1] onset, years: mean; SD; range)	37.8; 11.5; 17-57	35; 10.3; 18-54
F:M (n/n; ratio)	11/7; 1.6	14/4; 3.5
Duration of MS since diagnosis before DMF therapy initiation (months: mean; SD; range)	2; 0; 2	9.3; 8.1; 2-27
Previous therapies	None (18)	None (10), IFN-β 1a (6), Glatiramer acetate (2),
DMF treatment (months: mean; SD)	12; 0	10.8; 6.0
EDSS (mean; SD; range) before DMF therapy	0.8; 0.9; 0–2.5	1.7; 1.2; 0-4.0
EDSS (mean; SD; range) after DMF therapy	0.8; 0.9; 0–2.5	2.1; 1.3; 0-4.5

[1]: according to McDonald criteria (Polman et al., 2011)

Patient ID	Age at MS onset (years)	Gender	MS duration since diagnosis before DMF treatment (months)	Previous therapies	EDSS before DMF treatment	EDSS after DMF treatment	New/enlarging T2-weighted lesions in MRI under DMF treatment (y/n)	Relapse under DMF treatment (y/n)	DMF treatment (months)	% of $CD8^+CD45RA^-IL\text{-}17^+$ cells before DMF treatment	% of $CD8^+CD45RA^-IL\text{-}17^+$ cells after DMF treatment
1	17	F	2	0	0	0	n	n	12	3.2	1.2
2	22	F	2	0	1.5	1.5	n	n	12	1.4	1.0
3	24	F	2	0	2.5	2.5	n	n	12	1.3	0.3
4	31	F	2	0	1.5	1	n	n	12	1.4	0.5
5	31	F	2	0	1.5	1.5	n	n	12	0.7	0.0
6	34	F	2	0	1	1	n	n	12	0.7	0.2
7	37	F	2	0	1.0	1.0	n	n	12	3.2	0.8
8	42	F	2	0	0	0	n	n	12	2.1	1.6
19	42	F	2	0	0	0	n	n	12	0.6	0.2
10	43	F	2	0	0	0	n	n	12	2.0	0.3
11	49	F	2	0	1.5	1.5	n	n	12	0.6	0.3
12	57	F	2	0	0	0	n	n	12	0.3	0.1
13	28	M	2	0	0	0	n	n	12	0.6	0.7
14	31	M	2	0	2.5	2.5	n	n	12	0.4	0.2
15	41	M	2	0	0	0	n	n	12	0.4	0.3
16	45	M	2	0	0	0	n	n	12	0.0	0.3
17	51	M	2	0	0	0	n	n	12	2.6	0.7
18	56	M	2	0	1	1	n	n	12	0.6	0.4

Supplementary Table 2. Clinical characteristics of DMF responder MS patients, n=18. Responsiveness was defined as NEDA-3 positive status (i, no relapses, ii, no sustained disability progression measured with the expanded disability status scale (EDSS) and iii, no new/enlarging T2-weighted lesions in magnetic resonance imaging (MRI)) under an appropriate treatment period with DMF (re-baselining 4 months after treatment start).

Patient ID	Age at MS onset (years)	Gender	MS duration since diagnosis before DMF treatment (months)	Previous therapies	EDSS before DMF treatment	EDSS after DMF treatment	New/enlarging T2-weighted lesions in MRI under DMF treatment (y/n)	Relapse under DMF treatment (y/n)	DMF treatment (months)	% of $CD8^{+}CD45RA^{-}IL$-17^{+} cells before DMF treatment	% of $CD8^{+}CD45RA^{-}IL$-17^{+} cells after DMF treatment
1	26	F	2	0	1.0	1.5	n	y	6	0,0	0,1
2	28	F	2	0	1	1	y	n	12	0,5	0,3
3	33	F	2	0	1	1	y	n	5	0,4	0,8
4	34	F	2	0	1	1	y	n	11	0,1	0,1
5	45	F	3	0	2	2	y	n	26	0,3	0
6	28	F	5	0	0	0	y	y	9	0,4	0,1
7	18	F	8	0	0	0	y	n	11	0,1	0,3
8	33	F	19	0	1	2	n	y	14	0,2	0,1
9	26	M	2	0	1.0	1.0	y	n	8	0,1	0,3
10	22	M	3	0	1	2	y	y	24	0,3	0,2
11	38	F	11	IFN-β	2.5	2.5	y	y	6	0,1	0,2
12	45	F	12	IFN-β	2.0	2.5	y	y	12	0,3	0,4
13	45	F	23	IFN-β	2.0	2.5	y	y	6	0,3	0,3
14	54	F	27	IFN-β	4.0	4.5	y	y	14	0,5	0
15	32	F	9	IFN-β	2.0	2.5	y	y	10	0	1,3
16	54	M	21	IFN-β	3.5	4.0	y	y	5	0,7	0,9
17	39	F	10	Glatiramer acetate	3.5	4.0	y	y	11	0,4	0,1
18	30	F	6	Glatiramer acetate	3.0	3.5	n	y	5	0,2	0,1

Supplementary Table 3. Clinical characteristics of DMF non-responder MS patients, n=18. Non-responsiveness was defined as NEDA-3 negative status (either: i, relapse activity or ii, EDSS worsening or iii, new/enlarging T2-weighted lesions in MRI) under an appropriate treatment period with DMF (re-baselining 4 months after treatment start).

Supplementary Table 4. Characteristics of MS patient cohort included for transcriptome analysis (Fig 2f-l, Supplementary Fig. 2b-g).

	**DMF untreated**	**DMF treated**	
		Before	After
Age at MS[1] onset, (years: mean; SD; range)	30.6; 11.1; 20-46	37.3; 10.1; 23-45	37.3; 10.1; 23-45
F:M (n/n)	3/1	1/3	1/3
Duration of MS since diagnosis (years: mean; SD; range)	5.0; 10.0; 0-20	6.8; 7.3; 0-14	7.8; 7.3; 1-15
EDSS (mean; SD; range)	1.4; 1.1; 0-2.5	0.9; 1.1; 0-2.5	0.9; 1.1; 0-2.5
Previous therapies	None (3)<br>Glatiramer acetate, IFN-β 1a, Fingolimod (1)	None (2)<br>IFN-β 1a (2)	None (2)<br>IFN-β 1a (2)
DMF treatment<br>(months: mean; SD; range)	0; 0; 0	0; 0; 0	12; 0.8; 11-13

[1]: according to McDonald criteria (Polman et al., 2011)

Supplementary Table 5. Primers for ChIP analyses

**Primer**	**Sequences**
Il17a promoter	*5'-GAACTTCTGCCCTTCCCATCT -3'*<br>*5'-AGCACAGAACCACCCCTTT-3'*
Il17a -5	*5'-CGATACTTTTCAGTGACATCCGTTT-3'*<br>*5'-TGCTGACTTCATCTGATACCCTTAGA-3'*
RpL32 promoter	*5'-TCATTTCTCAGGCACATCTT-3'*<br>*5'-ACTCACCGTAAAACAGATGG-3'*
Il10 P2 promoter	5'-GCAGAAGTTCATTCCGACCA-3'<br>5'-GGCTCCTCCTCCCTCTTCTA-3'

Supplementary Table 6. Statistical evaluation of data presented in Main Figures.

Figure	test type	n (per group)	comparison	p value	T(df); F(Dfn, Dfd) or W(Dfn,Dfd)
1a 1st	two-tailed Paired t-test	18	responders: CD8 before vs after	0.0011	t(17)=3.934
1a 2nd	two-tailed Paired t-test	18	non-responders: CD8 before vs after	0.72	t(17)=0.3644
1a 3rd	two-tailed Paired t-test	18	responders: CD4 before vs after	0.3398	t(17)=0.9821
1a 4th	two-tailed Paired t-test	18	non-responders: CD4 before vs after	0.3499	t(17)=0.9613
1a $CD8^{+}CD45RA^{-}$	two-tailed, unpaired t-test	18	D treatment: Responder vs Non-Responder	0.0020	t(38)=3.322
1a $CD8^{+}CD45RA^{-}$	two-tailed, unpaired t-test	18	D treatment: Responder vs Non-Responder	0.6155	t(38)=0.5064
1b	two-tailed, unpaired t-test	18	CD8+CD45RA- Responder vs. Non-Responder before DMF therapy	0.003	t(34)=3.988
1c	One-way ANOVA + Tukey's HSD	5	DMSO vs. DMF<br>DMF vs. DMF+GSH<br>DMF vs. DMF+NAC	<0.0001<br>0.0008<br>0.0006	F(3,16)=17.17
1d	One-way Welsh ANOVA + Games-Howell	5	Ctrl vs. DMF<br>Ctrl vs. DMF+GSH<br>DMF vs. DMF+NAC	0.0005<br>0.0646<br>0.0356	W(2.000, 6.140)= 46.19
1e	One-way Welsh ANOVA + Games-Howell	7	Ctrl vs. DMF<br>Ctrl vs. DMF+GSH<br>DMF vs. DMF+NAC	0.0063<br>0.9983<br>0.0077	W(2,000, 11,97)= 13,86
1f	two-tailed, unpaired t-test with Welch's correction	4	Ctrl vs. DMF	0,0378	t(3)=3.560
1g	two-tailed, unpaired t-test	4	Ctrl vs. DMF	0.1970	t(10)=1.382
1h	two-tailed, unpaired t-test	9	Ctrl vs. DMF	0.0003	t(18)=4.472
1i	One-way ANOVA + Tukey's HSD	5	Ctrl vs DMF<br>Ctrl vs 2-DG<br>DMF vs 2-DG	0.0006<br>0.0007<br><0.0001	F (2,12)=53.24
1j	One-way ANOVA + Tukey's HSD	3	DMSO vs Rotenone<br>DMSO vs Oligomycin<br>DMSO vs DMF<br>DMSO vs DMF+GSH<br>DMF vs DMF+GSH	< 0.0001<br>< 0.0001<br>0.0003<br>0.0601<br>< 0.0001	F (4, 10) = 98.92
2b	One-way ANOVA + Tukey's HSD	3	contrl vs. DMF<br>contrl vs. DMF+GSH<br>DMF vs. DMF+GSH	0.0158<br>0.4982<br>0.0649	F (2, 6) = 8,671
2c	One-way ANOVA + Tukey's HSD	4	DMSO RORgt/Tbet vs. DMF RORgt/Tbet<br>DMSO RORgt/Tbet vs. DMF+GSH RORgt/Tbet<br>DMF RORgt/Tbet vs. DMF+GSH RORgt/Tbet	0.0008<br>0.5012<br>0.0004	F (2, 8) = 27,92
2i	two-tailed, unpaired t-test	4	DMF treated vs. untreated	0.0492	t(6)=2.459
3b	two-tailed, unpaired t-test	5	Ly294002 vs DMF+Ly294002	0.0258	t(7)=2.82
3c	One-way Welsh ANOVA + Games-Howell	6	DMSO vs DMSO+AKTi<br>DMSO vs DMF<br>DMSO vs DMF+AKTi<br>DMSO vs DMF+GSH<br>DMF vs DMF+AKTi<br>DMF vs DMF+GSH	0.0007<br>0.027<br>0.0321<br>0.9278<br>0.0055<br>0.0342	F (4, 19) = 27.74
3d	One-way ANOVA + Tukey's HSD	5	DMSO vs DMSO+AKTi<br>DMSO vs DMF<br>DMSO vs DMF+AKTi<br>DMSO vs DMF+GSH<br>DMF vs DMF+AKTi<br>DMF vs DMF+GSH	0.014<br>0.02<br>0.4515<br>>0.9999<br>0.0007<br>0.0154	F(4,19)=13.49
3e	One-way ANOVA + Tukey's HSD	4	Ctrl vs DMF<br>Ctrl vs DMF+GSH<br>DMF vs DMF+GSH	0.0106<br>0.9293<br>0.0062	F(2,9)=10.64
3g	two-tailed, unpaired t-test	4<br>4	WT Ctrl vs WT DMF<br>Tbx21KO Ctrl vs Tbx21KO DMF	0.0019<br>0.9785	t(4)=2.486
3h	two-tailed, unpaired t-test with Welch's correction	5<br>4	WT Ctrl vs WT DMF<br>Tbx21KO Ctrl vs Tbx21KO DMF	0.0271<br>0.2401	t(4)=3.408<br>t(3)=1.461
3i	two-tailed, unpaired t-test	4	WT vs Tbx21KO	0.0137	t(6)=3.444

Figure	test type	n (per group)	comparison	p value	T(df); F(Dfn, Dfd) or W(Dfn,Dfd)
4b	One-way ANOVA + Tukey's HSD	3	Ctrl 50 IL2 vs. DMF 50 IL2	0.0085	F (3,8) = 9.823
			Ctrl -IL2 vs. DMF-IL2	0.9092	
4c	One-way ANOVA + Tukey's HSD	3	0 DMSO vs. 0 DMF	0.9751	
			0 DMSO vs. 0 GSH	0.8745	F (2,28)= 0.1228
			0 DMF vs. 0 GSH	0.9402	
			10 DMSO vs. 10 DMF	0.2857	
			10 DMSO vs. 10 GSH	0.9469	F (2, 28) = 1.,655
			10 DMF vs. 10 GSH	0.3217	
			20 DMSO vs. 20 DMF	0.1199	
			20 DMSO vs. 20 GSH	0.9303	F (2, 19) = 2.294
			20 DMF vs. 20 GSH	0.4273	
			50 DMSO vs. 50 DMF	0.0007	
			50 DMSO vs. 50 GSH	0.9996	F (2, 31) = 10.25
			50 DMF vs. 50 GSH	0.0076	
			80 DMSO vs. 80 DMF	<0.0001	
			80 DMSO vs. 80 GSH	0.9103	F (2, 27) = 15.93
			80 DMF vs. 80 GSH	0.0007	
			150 DMSO vs. 150 DMF	<0.0001	
			150 DMSO vs. 150 GSH	0.8208	F (2, 26) = 39.83
			150 DMF vs. 150 GSH	<0.0001	
			500 DMSO vs. 500 DMF	0.1132	
			500 DMSO vs. 500 GSH	0.2668	F (2, 11) = 6.857
			500 DMF vs. 500 GSH	0.0107	
4d	One-way ANOVA + Tukey's HSD		+IL2 DMSO vs. +IL2 DMF	0.0004	
		5	+IL2 DMSO vs. +IL2 GSH	0.2153	F (2, 13) = 14.61
			+IL2 DMF vs. +IL2 GSH	0.0117	
			-IL2 DMSO vs. -IL2 DMF	0.0072	
		3	-IL2 DMSO vs. -IL2 GSH	0.1946	F (2, 8) = 9.059
			-IL2 DMF vs. -IL2 GSH	0.1566	
4e	One-way Welsh ANOVA + Games-Howell		DMSO vs. DMF	0.0048	
		6/7	DMSO vs. DMF +GSH	0.669	W(2.000, 6.667)=15.86
			DMF vs. DMF +GSH	0.002	
			DMSO -IL2 vs. DMF-IL2	0.0441	
		5	DMSO -IL2 vs. DMF+GSH -IL2	0.7168	W(2.000, 5.333)= 6.413
			DMF-IL2 vs. DMF+GSH -IL2	0.0691	
4f	One-way ANOVA + Tukey's HSD	3	wo DMSO vs. wo DMF	0.9506	
			wo DMSO vs. wo DMF+GSH	0.4545	F (2, 6) = 1.416
			wo DMF vs. wo DMF+GSH	0.3213	
			10 DMSO vs. 10 DMF	0.001	
			10 DMSO vs. 10 DMF+GSH	0.521	F (2, 6) = 38.97
			10 DMF vs. 10 DMF+GSH	0.0004	
			20 DMSO vs. 20 DMF	0.016	
			20 DMSO vs. 20 DMF+GSH	0.8781	F (2, 5) = 13.46
			20 DMF vs. 20 DMF+GSH	0.0108	
			40 DMSO vs. 40 DMF	0.0384	
			40 DMSO vs. 40 DMF+GSH	0.6298	F (2, 6) = 9.883
			40 DMF vs. 40 DMF+GSH	0.0129	
4g	two-tailed, unpaired t-test	3	pMIG empty vs pMIG STAT5	0.0006	t(6)=6.517
4h	One-way ANOVA + Tukey's HSD	6	0,2% DMSO vs. 20uM DMF	<0.0001	
			0,2% DMSO vs. DMF+35uM Inhibitor	<0.0001	F (2, 15) = 136.5
			20µM DMF vs. DMF+35µM Inhibitor	0.0001	

5a left	One-way ANOVA + Tukey's HSD	3	DMSO vs. DMF	0.0188	
			DMSO vs. DMF+GSH	0.9742	F (2, 9) = 7.349
			DMF vs. DMF+GSH	0.0263	
5a 2nd left	One-way ANOVA + Tukey's HSD	3	DMSO vs. DMF	0.0171	
			DMSO vs. DMF+GSH	0.5802	F (2, 6) = 8.529
			DMF vs. DMF+GSH	0.0586	
5a 3rd left	One-way ANOVA + Tukey's HSD	3	DMSO vs. DMF	0.0006	
			DMSO vs. DMF+GSH	0.0076	F (2, 6) = 30.94
			DMF vs. DMF+GSH	0.0498	
5a right	One-way ANOVA + Tukey's HSD	3	DMSO vs. DMF	0.0252	
			DMSO vs. DMF+GSH	0.3432	F (2, 6) = 6.679
			DMF vs. DMF+GSH	0.168	
5b left	One-way ANOVA + Tukey's HSD	3	DMSO vs. DMF	0.7905	
			DMSO vs. DMF+GSH	0.439	F (2, 6) = 2.029
			DMF vs. DMF+GSH	0.198	
5b right	One-way ANOVA + Tukey's HSD	3	DMSO vs. DMF	0.7735	
			DMSO vs. DMF+GSH	0.2071	F (2, 6) = 3.748
			DMF vs. DMF+GSH	0.0853	
5c left	One-way ANOVA + Tukey's HSD	3	DMSO vs. DMF	0.0045	
			DMSO vs. DMF+GSH	0.7227	F (2, 9) = 10.96
			DMF vs. DMF+GSH	0.0142	
5c right	One-way ANOVA + Tukey's HSD	3	DMSO vs. DMF	0.0561	
			DMSO vs. DMF+GSH	0.2041	F (2, 9) = 3.865
			DMF vs. DMF+GSH	0.6812	
5d	One-way ANOVA + Tukey's HSD	3	DMSO vs. DMF	0.2409	
			DMSO vs. DMF+GSH	0.8899	F (2, 6) = 1.794
			DMF vs. DMF+GSH	0.4177	

Figure	test type	n (per group)	comparison	p value	T(df); F(Dfn, Dfd) or W(Dfn,Dfd)
6a	Two-way ANOVA + Bonferroni	7	Interaction	<0.0001	F (26, 266) = 8,560
			time	<0.0001	F (13, 266) = 28,58
			treatment	<0.0001	F (2, 266) = 100,5
			d8 DMF - ctrl		
			0	>0,9999	
			5	>0,9999	
			6	>0,9999	
			7	>0,9999	
			8	>0,9999	
			9	>0,9999	
			10	0.5825	
			11	0.5825	
			12	0.3163	
			13	0.0014	
			14	0.0003	
			15	<0,0001	
			16	<0,0001	
			17	<0,0001	
6b	two-tailed, unpaired t-test with Welch's correction	7	ctrl vs DMF treated	0,0028	t(8.499)=4.164
6c	two-tailed, unpaired t-test with Welch's correction	7	ctrl vs DMF treated	0,0321	t(8.520)=2.558
6d	two-tailed, unpaired t-test	7	ctrl vs DMF treated	0,0335	t(11)=2.429
6e	Two-way ANOVA + Bonferroni	7	Interaction	<0.0001	F (22, 192) = 7.555
			Row Factor	<0.0001	F (11, 192) = 17.39
			Column Factor	<0.0001	F (2, 192) = 20.39
			d11		
			Irf4$^{-/-}$+2D2 vs. Irf4$^{-/-}$+2D2 + Tc17 DMSO	>0.9999	
			Irf4$^{-/-}$+2D2 vs. Irf4$^{-/-}$+2D2+Tc17 DMF	>0.9999	
			Irf4$^{-/-}$+2D2 + Tc17 DMSO vs. Irf4$^{-/-}$+2D2+Tc17 DMF	>0.9999	
			d12		
			Irf4$^{-/-}$+2D2 vs. Irf4$^{-/-}$+2D2 + Tc17 DMSO	>0.9999	
			Irf4$^{-/-}$+2D2 vs. Irf4$^{-/-}$+2D2+Tc17 DMF	>0.9999	
			Irf4$^{-/-}$+2D2 + Tc17 DMSO vs. Irf4$^{-/-}$+2D2+Tc17 DMF	>0.9999	
			d13		
			Irf4$^{-/-}$+2D2 vs. Irf4$^{-/-}$+2D2 + Tc17 DMSO	0.0848	
			Irf4$^{-/-}$+2D2 vs. Irf4$^{-/-}$+2D2+Tc17 DMF	>0.9999	
			Irf4$^{-/-}$+2D2 + Tc17 DMSO vs. Irf4$^{-/-}$+2D2+Tc17 DMF	0.2954	
			d14		
			Irf4$^{-/-}$+2D2 vs. Irf4$^{-/-}$+2D2 + Tc17 DMSO	<0.0001	
			Irf4$^{-/-}$+2D2 vs. Irf4$^{-/-}$+2D2+Tc17 DMF	>0.9999	
			Irf4$^{-/-}$+2D2 + Tc17 DMSO vs. Irf4$^{-/-}$+2D2+Tc17 DMF	<0.0001	
			d15		
			Irf4$^{-/-}$+2D2 vs. Irf4$^{-/-}$+2D2 + Tc17 DMSO	<0.0001	
			Irf4$^{-/-}$+2D2 vs. Irf4$^{-/-}$+2D2+Tc17 DMF	>0.9999	
			Irf4$^{-/-}$+2D2 + Tc17 DMSO vs. Irf4$^{-/-}$+2D2+Tc17 DMF	<0.0001	
6f	One-way Welsh ANOVA + Games-Howell	4-6	2D2 vs. Tc17 DMSO	0.0022	W (2.000, 6.811) = 22,46
			2D2 vs. Tc17 DMF	0.2948	
			Tc17 DMSO vs. Tc17 DMF	0.0147	
6j	One-way ANOVA + Dunett	4	DMSO CD4 vs. Ctrl CD4	0.1214	F (2, 10) = 2,305
			DMSO CD4 vs. DMF CD4	0.9451	
6j right	two-tailed, unpaired t-test	5	2D2+Tc17 vs 2D2+Tc17DMF	0.0178	t(6)=3.234
6k	One-way ANOVA + Dunett	7	DMSO CD4 vs. Ctrl CD4	0.0328	F (2, 17) = 5,803
			DMSO CD4 vs. DMF CD4	0.0133	
6k right	two-tailed, unpaired t test with Welch's correction	8	2D2+Tc17 vs 2D2+Tc17DMF	0.0481	t(7.758)=2.343

Supplementary Table 7. Statistical evaluation of data presented in Supplementary Figures.

Figure	test type	n (per group)	comparison	p value	T(df); F(Dfn, Dfd) or W(Dfn,Dfd)
1c	Two-tailed, unpaired	18	CD4+CD45RA- Responder vs. Non-Responder before DMF therapy	0.0914	t(1875)=1.780
S1d	One-way ANOVA + Tukey's HSD	7	DMSO vs. DMF	0.002	F(2,18)=11.36
			DMSO vs. DMF+GSH	0.9915	
			DMF vs. DMF+GSH	0.0015	
S1e	One-way ANOVA + Tukey's HSD	5	DMSO vs. DMF	0.0003	F(2,12)=19.85
			DMSO vs. DMF+GSH	0.9363	
			DMF vs. DMF+GSH	0.0005	
S1f	One-way ANOVA + Tukey's HSD	4	DMSO vs. DMSO+Trolox	0.9795	F (3, 10) = 12.01
			DMSO vs. DMF	0.0023	
			DMSO vs. DMF+Trolox	0.0452	
			DMF vs. DMF+Trolox	0.1689	
S1g	One-way ANOVA + Tukey's HSD	6	0,2% DMSO vs. 20µM DMF	0.9962	F (3, 20) = 18.10
			0,2% DMSO vs. 40µM DMF	<0.0001	
			0,2% DMSO vs. 40µM DMF+GSH	0.6931	
			20µM DMF vs. 40µM DMF	<0.0001	
			20µM DMF vs. 40µM DMF+GSH	0.8136	
			40µM DMF vs. 40µM DMF+GSH	0.0002	
S1h	One-way ANOVA + Tukey's HSD	3	DMSO vs. DMF	0.0673	F (2, 5) = 5.468
			DMSO vs. DMF+GSH	0.9984	
			DMF vs. DMF+GSH	0.1017	
S1i	Two-tailed, unpaired t-test	3-4	p1	0.0766	t(4)=2.373
			p2	0.0345	t(4)=3.152
			p3	0.014	t(4)=4.173
			p4	0.0054	t(4)=5.482
			p5	0.0027	t(4)=6.615
S1j	One-way ANOVA + Tukey's HSD	3	Ctrl vs. DMF	0.0077	F (2, 7) = 19.73
			Ctrl vs. DMSO + 2-DG	0.3746	
			DMF vs. DMSO + 2-DG	0.0012	
S3b	One-way ANOVA + Tukey's HSD	3	ctrl vs. DMF	0,716	F (3, 8)= 0,7896
			ctrl vs. AKTi	0,9629	
			ctrl vs. AKTi+DMF	0,0056	
			DMF vs. AKTi	0,9303	
			DMF vs. AKTi+DMF	0,0227	
			AKTi vs. AKTi+DMF	0,0103	
S3e	One-way ANOVA + Tukey's HSD	3	DMSO vs. DMF	0.0009	F (3, 6) = 171.7
			DMSO vs. DMF+Rapa50nM	<0.0001	
			DMSO vs. DMF+GSH	0.011	
			DMF vs. DMF+Rapa50nM	<0.0001	
			DMF vs. DMF+GSH	<0.0001	
			DMF+Rapa50nM vs. DMF+GSH	0.0004	
S3f	One-way ANOVA + Tukey's HSD	4	ctrl vs. DMF	0,5023	F (2, 9) = 9,426
			ctrl vs. Rapamycin	0,0058	
			DMF vs. Rapamycin	0,0339	
S3g	One-way ANOVA + Tukey's HSD	7	DMSO vs. DMF	0.0003	F (2, 18) = 15.33
			DMSO vs. Rapamycin 100/150nM	0.9297	
			DMF vs. Rapamycin 100/150nM	0.0006	
S4b	Two-tailed, Unpaired t-test	4	GFP neg	0.1346	t(6)=1.728
			GFP low	0.0069	t(6)=4.032
			GFP int	0.0011	t(6)=5.818
S5b	One-way ANOVA + Tukey's HSD	6	DMSO vs. DMF	0.0007	F (2, 14) = 11.94
			DMSO vs. DMF+TSA	0.1293	
			DMF vs. DMF+TSA	0.0548	
S5d left	One-way ANOVA + Tukey's HSD	4	DMSO vs. DMF	0.3105	F (2, 9) = 1.225
			DMSO vs. DMF+GSH	0.6608	
			DMF vs. DMF+GSH	0.785	
S5d right	One-way ANOVA + Tukey's HSD	5	DMSO vs. DMF	0.5866	F (2, 12) = 1.200
			DMSO vs. DMF+GSH	0.3153	
			DMF vs. DMF+GSH	0.8652	
S5e	One-way ANOVA + Tukey's HSD	4	DMSO vs. DMF	0.9666	F (2, 9) = 0.1818
			DMSO vs. DMF+GSH	0.8236	
			DMF vs. DMF+GSH	0.9347	
S6c	Two-way ANOVA + Bonferroni	8	Interaction	<0.0001	F (14, 207) = 6.409
			Row Factor	<0.0001	F (14, 207) = 37.51
			Column Factor	<0.0001	F (1, 207) = 34.53
			Water - DMF		
			Row 1	>0.9999	
			Row 2	>0.9999	
			Row 3	>0.9999	
			Row 4	>0.9999	
			Row 5	>0.9999	
			Row 6	>0.9999	
			Row 7	>0.9999	
			Row 8	>0.9999	
			Row 9	>0.9999	
			Row 10	>0.9999	
			Row 11	>0.9999	
			Row 12	0.2495	
			Row 13	<0.0001	
			Row 14	<0.0001	
			Row 15	<0.0001	

S6d	Two-tailed, Unpaired t test with Welch's correction	8	0.0488	t(8.090)=2.317
