# Supplementary material for: IL-17+ CD8+ T cell suppression by dimethyl fumarate associates with clinical response in multiple sclerosis
Source: Nat Commun. 2019 Dec 16;10:5722. doi: 10.1038/s41467-019-13731-z (PMC6915776; doi:10.1038/s41467-019-13731-z)
Supplement: Supplementary file 2 — Reporting Summary [file 41467_2019_13731_MOESM2_ESM.pdf]

nature research

Corresponding author(s): Magdalena Huber

Last updated by author(s): Nov 21, 2019

Reporting Summary

Nature Research wishes to improve the reproducibility of the work that we publish. This form provides structure for consistency and transparency in reporting. For further information on Nature Research policies, see Authors & Referees and the Editorial Policy Checklist.

Statistics

For all statistical analyses, confirm that the following items are present in the figure legend, table legend, main text, or Methods section.

n/a	Confirmed	
☐	☒	The exact sample size (n) for each experimental group/condition, given as a discrete number and unit of measurement
☐	☒	A statement on whether measurements were taken from distinct samples or whether the same sample was measured repeatedly
☐	☒	The statistical test(s) used AND whether they are one- or two-sided<br>*Only common tests should be described solely by name; describe more complex techniques in the Methods section.*
☒	☐	A description of all covariates tested
☐	☒	A description of any assumptions or corrections, such as tests of normality and adjustment for multiple comparisons
☐	☒	A full description of the statistical parameters including central tendency (e.g. means) or other basic estimates (e.g. regression coefficient) AND variation (e.g. standard deviation) or associated estimates of uncertainty (e.g. confidence intervals)
☐	☒	For null hypothesis testing, the test statistic (e.g. F, t, r) with confidence intervals, effect sizes, degrees of freedom and P value noted<br>*Give P values as exact values whenever suitable.*
☒	☐	For Bayesian analysis, information on the choice of priors and Markov chain Monte Carlo settings
☒	☐	For hierarchical and complex designs, identification of the appropriate level for tests and full reporting of outcomes
☐	☒	Estimates of effect sizes (e.g. Cohen's d, Pearson's r), indicating how they were calculated

Our web collection on statistics for biologists contains articles on many of the points above.

Software and code

Policy information about availability of computer code

Data collection	Data collection is described in detail in the methods section and using the following devices:<br>Flow Cytometry cell sorter ARIA III (BD), Flow Cytometry analyser Attune (Thermofischer),<br>XF-96 Extracellular Flux Analyzer (Agilent), Illumina HiSeq2500,
Data analysis	Data analysis is described in detail in the methods section,, also the software used, including lowJo V10.6.1, Diva 8, GraphPad Prism8;

For manuscripts utilizing custom algorithms or software that are central to the research but not yet described in published literature, software must be made available to editors/reviewers. We strongly encourage code deposition in a community repository (e.g. GitHub). See the Nature Research guidelines for submitting code & software for further information.

Data

Policy information about availability of data

All manuscripts must include a data availability statement. This statement should provide the following information, where applicable:

- Accession codes, unique identifiers, or web links for publicly available datasets
- A list of figures that have associated raw data
- A description of any restrictions on data availability

The murine Affymetrix microarray data is available under GSE110346.
The murine RNA-seq data has been deposited to the GEO repository under accession number GSE116866.
The human data of MS patients treatment is available under GSE116865.
The other data that support the findings of this study are available from the corresponding author upon reasonable request.

Field-specific reporting

Please select the one below that is the best fit for your research. If you are not sure, read the appropriate sections before making your selection.

☒ Life sciences ☐ Behavioural & social sciences ☐ Ecological, evolutionary & environmental sciences

For a reference copy of the document with all sections, see nature.com/documents/nr-reporting-summary-flat.pdf

Life sciences study design

All studies must disclose on these points even when the disclosure is negative.

Sample size	The study design is described detailed in the method section.
Data exclusions	The exclusion parameters were indicated in the supplemental materials.
Replication	Number of repat are indicated on the figure legends. Experiments were analyzed using a minimum of three independent biological replicates
Randomization	The study design is described detailed in the method section.
Blinding	Investigators were blinded to treatment groups.

Reporting for specific materials, systems and methods

We require information from authors about some types of materials, experimental systems and methods used in many studies. Here, indicate whether each material, system or method listed is relevant to your study. If you are not sure if a list item applies to your research, read the appropriate section before selecting a response.

Materials & experimental systems

n/a	Involved in the study	
☐	☒	Antibodies
☒	☐	Eukaryotic cell lines
☒	☐	Palaeontology
☐	☒	Animals and other organisms
☐	☒	Human research participants
☒	☐	Clinical data

Methods

n/a	Involved in the study	
☒	☐	ChIP-seq
☐	☒	Flow cytometry
☒	☐	MRI-based neuroimaging

Antibodies

Antibodies used	detalied description of all antibodies is indicated in the method section
Validation	All antibodies were used in accordance with the manufacturers instructions and guidelines on their website.

Animals and other organisms

Policy information about studies involving animals; ARRIVE guidelines recommended for reporting animal research

Laboratory animals	All mice were 8-12 weeks old, at C57BL/6 background and sex- and age-matched. Further details are reported in the methods section of the manuscript.
Wild animals	N/A
Field-collected samples	N/A
Ethics oversight	Mouse experiments were approved by the local committee (Regierungspräsidium Gießen).

Note that full information on the approval of the study protocol must also be provided in the manuscript.

Human research participants

Policy information about studies involving human research participants

Population characteristics	The patient cohort and study design are described in detail in the methods section

Recruitment	The samples were recruited at the university of Mainz and Marburg. Details are indicated in the methods section.
Ethics oversight	The study was conducted according the rules of the Declaration of Helsinki. The Ethics Committees of the Universities of Mainz and Marburg provided approval for this study and blood samples blood was drawn after written informed consent was obtained.

Note that full information on the approval of the study protocol must also be provided in the manuscript.

Flow Cytometry

Plots

Confirm that:

☒ The axis labels state the marker and fluorochrome used (e.g. CD4-FITC).

☒ The axis scales are clearly visible. Include numbers along axes only for bottom left plot of group (a 'group' is an analysis of identical markers).

☒ All plots are contour plots with outliers or pseudocolor plots.

☒ A numerical value for number of cells or percentage (with statistics) is provided.

Methodology

Sample preparation	the preparation of the murine and human samples and further processing is described in detail inthe methods section
Instrument	for sorting and analysis: Aria III (BD) and Attune (Thermofischer)
Software	Diva V8 and FlowJo V10.06.1
Cell population abundance	The purity of the sorted populations was >97% in post sort analysis.
Gating strategy	For all experiments FSC-A/ SSC-A gates of the starting cell population were used followed by doublet exclusion using FSC-H/ FSC-A gating and live/dead discrimination. Further details of the gating strategies are described in methods section.

☒ Tick this box to confirm that a figure exemplifying the gating strategy is provided in the Supplementary Information.
